# Rapidly Degrading Hydrogels to Support Biofabrication and 3D Bioprinting Using Cartilage Microtissues

**DOI:** 10.1021/acsbiomaterials.4c00819

**Published:** 2024-09-06

**Authors:** Gabriela S. Kronemberger, Francesca D. Spagnuolo, Aliaa S. Karam, Kaoutar Chattahy, Kyle J. Storey, Daniel J. Kelly

**Affiliations:** † Trinity Centre for Biomedical Engineering, Trinity Biomedical Sciences Institute, Trinity College Dublin, Dublin D02 R590, Ireland; ‡ Department of Mechanical, Manufacturing and Biomedical Engineering, School of Engineering, Trinity College Dublin, Dublin D02 R590, Ireland; § Department of Anatomy and Regenerative Medicine, Royal College of Surgeons in Ireland, Dublin D02 YN77, Ireland; ∥ Advanced Materials and Bioengineering Research Centre (AMBER), Royal College of Surgeons in Ireland and Trinity College Dublin, Dublin D02 F6N2, Ireland

**Keywords:** microtissues, 3D bioprinting, support hydrogel, biofabrication, extrusion-based printing, fusion, cartilage

## Abstract

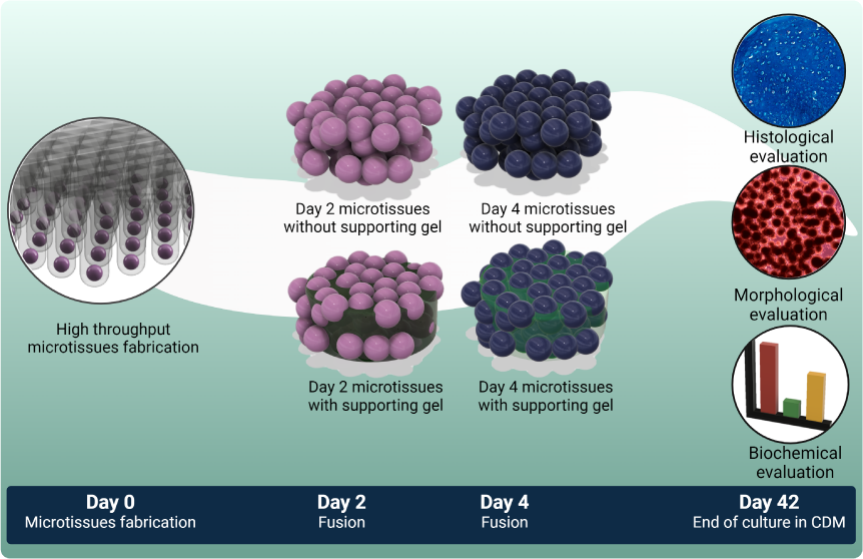

In recent years, there has been increased interest in the use of cellular spheroids, microtissues, and organoids as biological building blocks to engineer functional tissues and organs. Such microtissues are typically formed by the self-assembly of cellular aggregates and the subsequent deposition of a tissue-specific extracellular matrix (ECM). Biofabrication and 3D bioprinting strategies using microtissues may require the development of supporting hydrogels and bioinks to spatially localize such biological building blocks in 3D space and hence enable the engineering of geometrically defined tissues. Therefore, the aim of this work was to engineer scaled-up, geometrically defined cartilage grafts by combining multiple cartilage microtissues within a rapidly degrading oxidized alginate (OA) supporting hydrogel and maintaining these constructs in dynamic culture conditions. To this end, cartilage microtissues were first independently matured for either 2 or 4 days and then combined in the presence or absence of a supporting OA hydrogel. Over 6 weeks in static culture, constructs engineered using microtissues that were matured independently for 2 days generated higher amounts of glycosaminoglycans (GAGs) compared to those matured for 4 days. Histological analysis revealed intense staining for GAGs and negative staining for calcium deposits in constructs generated by using the supporting OA hydrogel. Less physical contraction was also observed in constructs generated in the presence of the supporting gel; however, the remnants of individual microtissues were more observable, suggesting that even the presence of a rapidly degrading hydrogel may delay the fusion and/or the remodeling of the individual microtissues. Dynamic culture conditions were found to modulate ECM synthesis following the OA hydrogel encapsulation. We also assessed the feasibility of 3D bioprinting of cartilage microtissues within OA based bioinks. It was observed that the microtissues remained viable after extrusion-based bioprinting and were able to fuse after 48 h, particularly when high microtissue densities were used, ultimately generating a cartilage tissue that was rich in GAGs and negative for calcium deposits. Therefore, this work supports the use of OA as a supporting hydrogel/bioink when using microtissues as biological building blocks in diverse biofabrication and 3D bioprinting platforms.

## 1 Introduction

Osteoarthritis is a musculoskeletal disease that affects more than 300 million patients worldwide.^1^ It is characterized by progressive degeneration of articular cartilage, subchondral bone, and other synovial structures,^2^ resulting in chronic pain and impaired quality of life.^3^ Classic tissue engineering strategies targeting this clinical problem have typically focused on the development of scaffolds seeded with chondrocytes or mesenchymal stem/stromal cells (MSCs);^4−6^ however, such approaches typically fail to generate tissues truly mimetic of articular cartilage, which may explain their poor regenerative outcomes *in vivo*.^7,8^ In recent years, there has been increased interest in the use of cellular spheroids, microtissues, or organoids as biological building-blocks for the biofabrication of complex tissues and organs.^9^ Microtissues are typically generated from cellular aggregates formed by a self-assembly process.^10^ The capacity of microtissues to fuse to one another can be leveraged to engineer larger, scaled-up tissues, processes that are often enabled by emerging biofabrication and 3D bioprinting strategies.^11−16^ Others have combined microtissues with polymeric microparticles carrying chondrogenic factors to generate cartilage-like constructs.^17^ In many cases, biofabrication using microtissues may require the development of support hydrogels and bioinks to spatially localize such biological building blocks in 3D space, thereby enabling the engineering of geometrically defined tissues.^18,19^

We have previously demonstrated that it is possible to engineer functional cartilage constructs by using 3D-printed polymeric scaffolds such as polycaprolactone (PCL) to guide microtissue fusion and subsequent ECM production and organization.^20−22^ One drawback related to this strategy is that PCL degrades slowly *in vivo*,^23^ and the long-term impact of implanting a biomaterial with the potential to promote fibrosis^24^ remains unclear. It has also been possible to engineer completely *scaffold-free* cartilage-like constructs using rapidly degrading hydrogels such as Pluronic F-127^25,26^ and gelatin^27,28^ that fully degrade *in vitro*. One particularly promising hydrogel for such applications is oxidized alginate (OA),^29^ which we have shown supports chondrogenesis of MSCs^30^ and can be used as a temporary supporting hydrogel for inkjet printing of cell suspensions.^31^ This suggests that OA might also be suitable as a temporary supporting hydrogel for the biofabrication of geometrically defined constructs using cartilage microtissues as biological building blocks or as a bioink for extrusion-based bioprinting of microtissues. Therefore, the aim of this work was to engineer scaled-up, geometrically defined cartilage grafts by combining multiple cartilage microtissues within a rapidly degrading oxidized alginate (OA) support hydrogel/bioink. We first explored the fusion capacity of MSC-derived microtissues in the presence and absence of a supporting OA hydrogel in both static and dynamic culture. To this end, cartilage microtissues were first independently matured for either 2 or 4 days and then combined in the presence or absence of a supporting OA hydrogel. Furthermore, we explored the 3D bioprinting of these microtissues using extrusion-based deposition in OA-based bioinks and assessed their capacity to fuse and generate cartilage-specific ECM postprinting. Our findings support the use of OA as a temporary support structure when microtissues are used as biological building blocks in diverse biofabrication and 3D bioprinting platforms.

## 2 Material and Methods

### 2.1 Bone Marrow Mesenchymal Stem/Stromal Cell (MSC) Isolation and Expansion

MSCs were isolated from the sternum of skeletally mature, female, Saanen goats as described previously.^21,22^ Briefly, after the bone marrow pieces were harvested, they were gently cut into small pieces by using a 10A scalpel. Next, the marrow pieces obtained were vortexed for 5 min in expansion medium (X-Pan) made from Dulbecco’s modified Eagle’s medium (DMEM) + 100 U/mL penicillin +100 μg/mL streptomycin (Gibco) + 10% (w/v) fetal bovine serum (FBS) + basic fibroblastic growth factor 2 (FGF-2) to aid in liberating the cellular components. Then, the culture medium containing the cell suspension was aspirated and passed through a 40 μm cell strainer prior to counting and plating at a density of 5.7 × 10^4^ cells/cm^2^ and expanded under physioxic conditions (37 °C in a humidified atmosphere with 5% CO_2_ and 5% O_2_) for improved chondrogenic differentiation. When the confluency in the flasks was at 80%, MSCs were trypsinized using 0.25% (w/v) trypsin ethylenediaminetetraacetic acid (EDTA). For microtissue culture, MSCs were expanded from an initial density of 5,000 cells/cm^2^ in X-Pan medium under physioxic conditions until passage 3.

### 2.2 Microtissue Fabrication and Differentiation

MSC-derived microtissues were fabricated using an in-house high-throughput nonadherent agarose hydrogel microwell system as described previously.^15,18^ In this system, it is possible to fabricate up to 1,880 microtissues at once. For microtissue formation, MSCs at passage 3 were seeded on top of each mold at a final density of 4 × 10^3^ cells/microtissue in X-Pan media. After 24 h, in order to allow cell aggregation, the microtissues were maintained in chondrogenic culture conditions consisting of hgDMEM GlutaMAX supplemented with 100 U/mL penicillin, 100 μg/mL streptomycin (both Gibco), 100 μg/mL sodium pyruvate, 40 μg/mL l-proline, 50 μg/mL l-ascorbic acid-2-phosphate, 4.7 μg/mL linoleic acid, 1.5 mg/mL bovine serum albumin, 1× insulin–transferrin–selenium (ITS), 100 nM dexamethasone (all from Sigma), 2.5 μg/mL amphotericin B, and 10 ng/mL of human transforming growth factor-beta 3 (TGF-β) (Peprotech, UK). The microtissues were cultured under physioxic conditions (37 °C in a humidified atmosphere with 5% CO_2_ and 5% O_2_) for up to 4 days.

### 2.3 Microtissue Diameter and Sphericity Measurements

MSC-derived microtissues were monitored, and images were acquired using the 4× objective of an optical inverted microscope (Primo Vert, Zeiss, USA) equipped with a digital camera. Width and length were measured using ImageJ 1.53e software (Wayne Rasband and Contributors, USA). A diameter ratio of each microtissue was obtained by dividing the width by length (sphericity). Three independent measurements of 15 spheroids were made from each experimental group.

### 2.4 OA Hydrogel/Bioink Development

The OA hydrogel was synthesized as described before.^29,30^ Briefly, 1 g of MVG sodium alginate (Pronova Biopolymers, Halland, Sweden) was dissolved in 90 mL of ultrapure deionized water overnight at 37 °C and mixed with 10 mL of sodium periodate (Honeywell, Charlotte, NC, USA) at a final concentration of 4%. The solution was continuously stirred in the dark at room temperature for 24 h, followed by a dialysis step against deionized water for 3 days (MWCO 3,500 Da; Fisher, Waltham, MA, USA). Next, the solution was sterile filtered through a 0.22 μm filter and lyophilized afterward.

To develop a printable bioink, gelatin was combined with the 4% (w/v) OA hydrogel to modify its overall viscosity. It was prepared using gelatin from bovine skin (Sigma) dissolved in hgDMEM GlutaMAX at a final concentration of 5% (w/v).

### 2.5 Rheological Assessment of Bioinks

The rheological properties of all bioinks and bioink components were assessed using a rheometer (MCR 102, Anton-Paar, Dublin, Ireland) equipped with a Peltier element for temperature control. A plate–plate geometry with a diameter of 25 mm (PP25) was used for all tests. Viscosity as a function of shear rate (0.1–1000 s^–1^) was measured at a constant temperature of 4 °C. The gelation kinetics of the bioinks and bioink components were assessed using a temperature sweep test from 4 to 37 °C with an increment of 1 °C every 30 s while maintaining a shear rate at 1 s^–1^.

### 2.6 Microtissues Encapsulation in the OA Hydrogel in Static and Dynamic Conditions

To evaluate the capacity of microtissues to fuse in the OA, a total of 1,000 microtissues encapsulated in the hydrogel (4% w/v) were cast into agarose molds. The agarose mold was prepared in 12 well plates by using a solution of 2% (w/v) agarose (Sigma) diluted in deionized water. The size of the agarose well was 3 mm in diameter and 1.5 mm in depth.^15^

For culture in static conditions, microtissues at day 2 and day 4 maturation levels were harvested, counted, and encapsulated manually in 4% (w/v) OA. 12 h prior to seeding, the agarose wells were soaked in 60 mM CaCl_2_ (Sigma) prepared in hgDMEM GlutaMAX, to obtain efficient cross-linking of the OA. After the microtissues were seeded in the agarose mold, 2 mL of CaCl_2_ was added to each well, and the constructs were maintained for 30 min in physioxic conditions at 37 °C for 30 min. After cross-linking, the CaCl_2_ was removed, and 3 mL of chondrogenic media was added to each well. As a control group, microtissues at the same maturation levels were seeded in the agarose mold without any supporting OA hydrogel. The constructs were then cultured in physioxic conditions at 37 °C for up to 6 weeks (Graphical abstract). Media exchange was performed thrice weekly until the end of the culture period. The constructs were fabricated in triplicates, and three independent experiments were performed.

For culture in dynamic conditions, microtissues at the day 2 maturation level were harvested and encapsulated in OA as described before. As a control group, microtissues at the same maturation level were seeded in the agarose mold without any supporting OA hydrogel. The dynamic culture involved the continuous linear actuation of the stage, over 5 mm of travel at a frequency of 0.048 Hz^22,32^^22^ for up to 6 weeks in physioxic conditions at 37 °C (Graphical abstract). Media exchange was performed thrice weekly until the end of the culture period. The constructs were fabricated in triplicate, and two independent experiments were performed.

### 2.7 Histological Evaluation

Briefly, samples were fixed using a 4% (w/v) paraformaldehyde (PFA) solution (Sigma) overnight at 4 °C. After fixation, samples were dehydrated in a graded series of ethanol solutions (70–100%), cleared in xylene, and embedded in paraffin wax (all Sigma). Tissue sections (5 μm) were taken at the center of the sample in the transverse plane using a manual microtome (Leica) and rehydrated before staining. Sections were stained with hematoxylin and eosin (HE) for morphology evaluation, 1% (w/v) Alcian Blue 8GX in 0.1 M hydrochloric acid (HCl) (AB) to visualize sulfated glycosaminoglycan (sGAG) and counter-stained with 0.1% (w/v) nuclear fast red to determine cellular distribution, 0.1% (w/v) picrosirius red (PSR) for collagen deposition, and 1% (w/v) alizarin red (pH 4.1) to identify calcium deposition (all from Sigma). Stained sections were then imaged using an Aperio ScanScope slide scanner (Leica).

### 2.8 Immunohistochemistry Analysis

Immunohistochemistry analyses were performed in the constructs at the end of the culture period to assess the chondrogenesis efficiency. The analyses were performed for collagen I (Abcam ab90395 1:400), collagen II (Santa Cruz sc52658 1:400), and collagen X (Abcam ab49945 1:200) as previously described.^15^

### 2.9 Biochemical Analysis

After being harvested, samples were washed two times in phenol-free DMEM (pfDMEM, Sigma-Aldrich, Wicklow, Ireland) and manually dried. Next, 3.88 U/mL of papain enzyme in 100 mM sodium phosphate buffer/5 mM Na_2_EDTA/10 mM l-cysteine, pH 6.5 (all from Sigma–Aldrich), was used to digest the samples at 60 °C for 18 h using a rotator. The completely digested samples were then vortexed for 1 min and centrifuged for 5 min at 650*g*.

The DNA content was quantified immediately after digestion using the Quant-iT PicoGreen dsDNA Reagent and Kit (Molecular Probes, Biosciences) following the company’s recommended steps. The DNA content of each sample was quantified using the Synergy HT multidetection microplate reader (BioTek Instruments, Inc.) at a wavelength of 480 nm.

The amount of glycosaminoglycans (GAGs) was determined using the dimethylmethylene (DMMB) blue dye-binding assay (Sigma), with a chondroitin sulfate solution (Sigma) for the standards. The pH of the DMMB was adjusted to 1.5 using a 12 N HCl solution. The samples were read using the Synergy HT multidetection microplate reader (BioTek Instruments, Inc.) at wavelengths of 530 and 590 nm.

The total collagen content was obtained by using a chloramine-T assay. Briefly, samples were initially mixed with 38% HCl (Sigma) and incubated at 110 °C for 18 h to allow hydrolysis to occur. Samples were subsequently dried in a fume hood, and the sediment was reconstituted in ultrapure water. A concentration of 2.82% (w/v) chloramine-T and 0.05% (w/v) 4-(dimethylamino) benzaldehyde (both Sigma) were then added to the samples, and the hydroxyproline content was quantified with a *trans*-4-hydroxy-l-proline (Fluka Analytical) standard using a Synergy HT multidetection microplate reader at a wavelength of 570 nm (BioTek Instruments, Inc.).

### 2.10 Extrusion-Based Bioprinting of Microtissues

MSC microtissues were 3D bioprinted by using a pneumatic extrusion-based technique on a BioX6 system (CellInk, Sweden). Initially, different densities (2,000, 4,000, and 10,000) of microtissues were combined with the OA and gelatin hydrogels. As mentioned before, gelatin was used as a carrier to increase the printability of the OA-based bioink. After preparation, the bioinks were loaded into a syringe kept at 4 °C for up to 15 min before starting the bioprinting process. Next, the syringes were placed in the printheads, and a plastic 16G conical needle (CellInk, Sweden) was used for printing. The microtissue constructs were then bioprinted at 14 °C, using a pressure range of 75–90 kPa at a speed of 4 mm/s following a grid design on air and/or inside the agarose wells used for the casting studies. The grid pattern had a strand distance of 0.500 mm. After 3D bioprinting, the constructs were incubated for 30 min in a bath of 60 mM CaCl_2_ prepared in hgDMEM GlutaMAX under physioxic conditions at 37 °C. After that period, the CaCl_2_ was manually removed, and the constructs were maintained in chondrogenic induction under physioxic conditions at 37 °C for up to 6 weeks. Media exchange was performed thrice weekly until the end of the culture period.

### 2.11 Viability Assay

For cell viability analyses of the bioprinted constructs after 10 days of culture, a LIVE/DEAD Cell Fluorescence assay was used. The assay is based on the use of calcein acetoxymethyl (Calcein-AM) and ethidium homodimer (EthD-1) (both from Biosciences, Dublin, Ireland) solutions, which stain viable and dead cells, respectively. Briefly, the bioprinted constructs were washed three times in phenol-free DMEM (pfDMEM, Sigma-Aldrich, Wicklow, Ireland) followed by incubation in pfDMEM containing 2 μM calcein AM and 4 μM EthD-1 for 1 h. Next, the constructs were washed three times in pfDMEM before imaging with a Leica SP8 scanning confocal microscope (Wetzlar, Germany) excited at 494 and 528 nm and read at 517 and 617 nm.

### 2.12 Statistical Analysis

Statistical analysis was performed using GraphPad Prism software (GraphPad Software, CA, USA). A one-way analysis of variance (ANOVA) was performed followed by Kruskal–Wallis multiple comparisons post-test to assess the differences in diameter and sphericity between the groups. For biochemical analysis, two-way ANOVA was performed followed by Tukey’s multiple comparison post-test to assess differences between groups. Numerical and graphical results are presented as the mean ± standard deviation. Significance was determined when *p<* 0.05.

## 3 Results

### 3.1 MSC-Derived Microtissues Generate a Cartilage-like Tissue following Encapsulation in a Temporary Supporting Hydrogel

**Figure 1:**
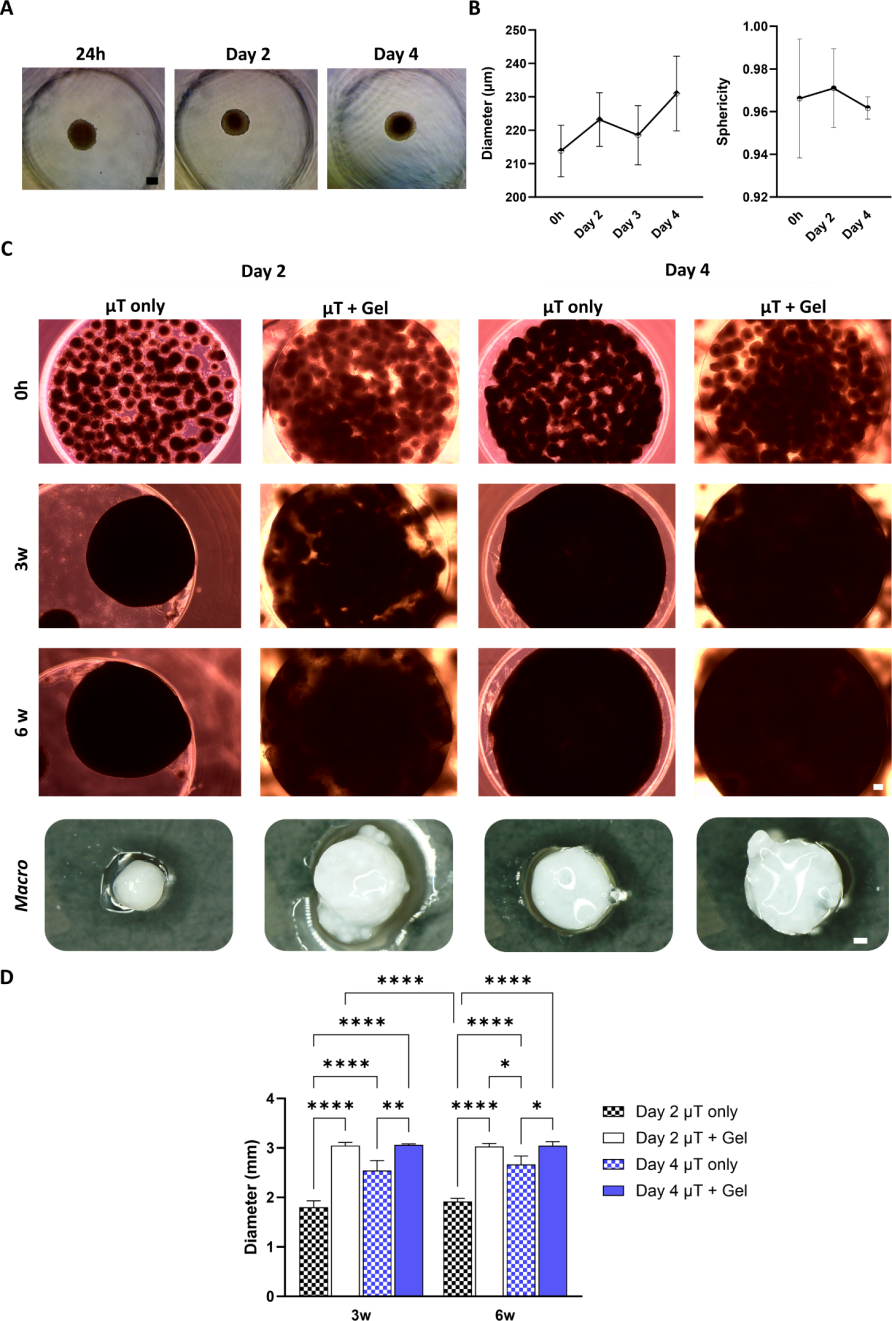
MSC microtissues show fusion capacity in the OA hydrogels. (A) Phase contrast of single MSC microtissues at 24 h, day 2, and day 4 of culture in chondrogenic medium. (B) Diameter and sphericity measurements of MSC microtissues over 4 days of culture in chondrogenic medium. (C) Phase contrast of day 2 and day 4 MSC-derived microtissues seeded in the agarose well, in the absence of the supporting hydrogel (μT only) or encapsulated in the OA (μT + Gel), at 0 h, 3, and 6 weeks. The bottom panel provides macroscopic images of the microtissues cultured in the absence of the temporary supporting hydrogel and microtissues encapsulated in the OA after 6 weeks of chondrogenic induction. (D) Diameter measurements of constructs generated using the day 2 and day 4 microtissues seeded in the agarose well, either in the absence of the supporting hydrogel (μT only) or encapsulated in the OA (μT + Gel), after 3 and 6 weeks of culture. The data are expressed as mean ± SD. The asterisks indicate *p*-values obtained by nonpaired *one-way* ANOVA followed by Kruskal–Wallis multiple comparisons (*p < 0.05; **p < 0.001; ****p < 0.0001). Scale bar: 100 μm.

Initially, the diameter and sphericity of MSC-derived microtissues were investigated over 4 days of chondrogenic culture (Figure 1A–C). Microtissues were formed using our in-house high-throughput agarose mold system^16^ and cultured in their individual microwells (Figure 1A). The average diameter of the microtissues increased from 210 to 250 μm over the first 4 days of culture (Figure 1B). However, at 0 h, day 2, and day 4 time points, similar sphericity values were observed, demonstrating the generation of microtissues with a relatively homogeneous shape (Figure 1B).

After either 2 or 4 days of maturation, the microtissues were removed from their individual microwells and either encapsulated in OA hydrogels (termed the “μT + Gel” group) or allowed to fuse in a supporting mold in the absence of any supporting hydrogel (termed the “μT only” group). As expected, in the absence of any supporting hydrogel, both day 2 and day 4 microtissues were able to fuse and generate a tissue that macroscopically appeared cartilage-like over 6 weeks in culture (Figure 1C). The microtissues also appeared to fuse and generate cartilage-like tissue when encapsulated into the OA hydrogels (Figure 1C). Less contraction of the overall tissue was observed in the presence of the supporting OA gel, with the final tissue better approximating the target geometry defined by the outer mold (diameter = 3 mm; Figure 1C,D). The diameter of the constructs generated in the absence of the supporting OA gel was significantly lower after both 3 and 6 weeks of culture (Figure 1D). This supports the hypothesis that when cartilage microtissues are encapsulated in OA, the resulting constructs better retain the initial shape defined by the external mold.

**Figure 2:**
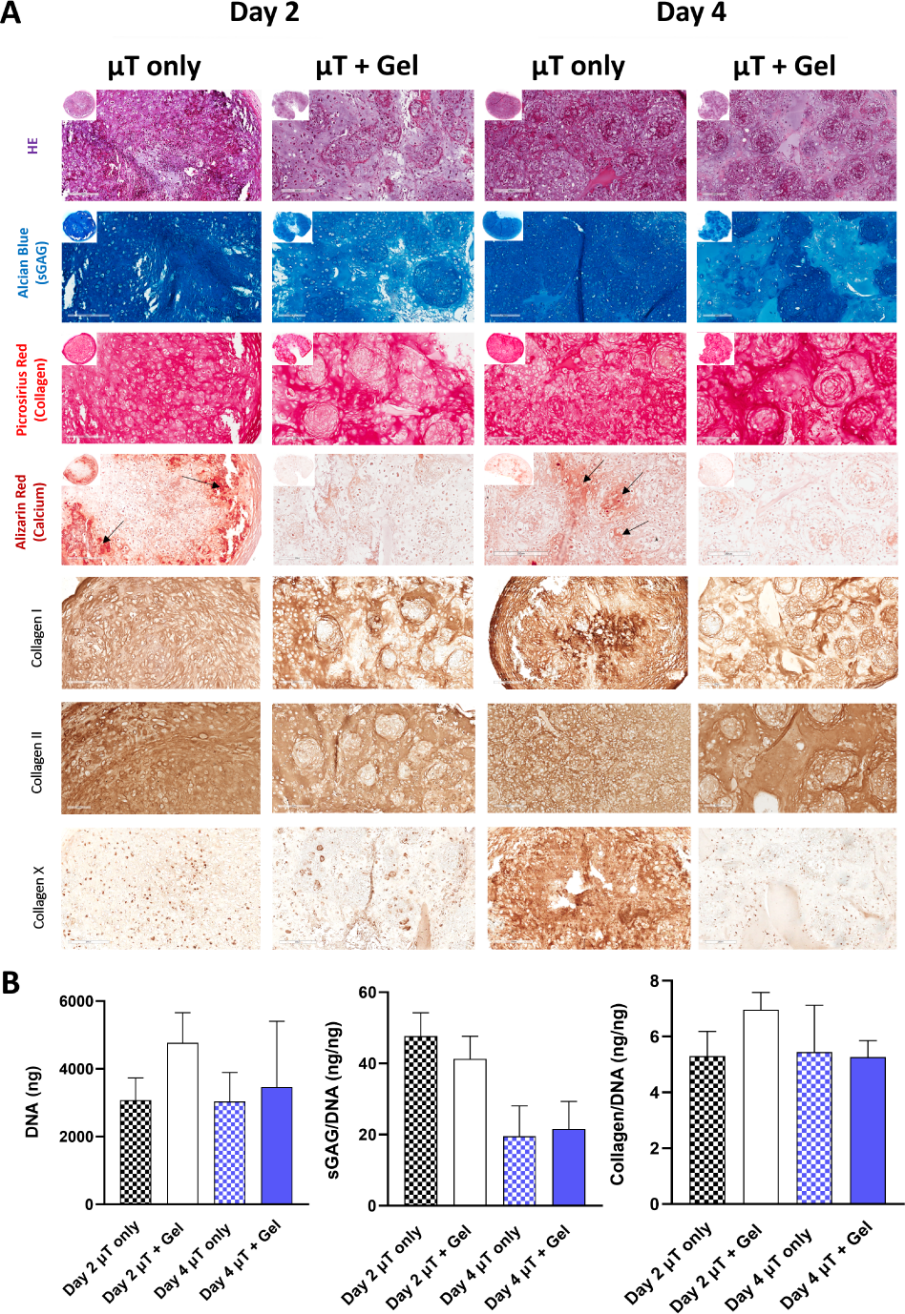
Chondrogenesis of MSC-derived microtissues following encapsulation in OA hydrogels. (A) Hematoxylin and eosin, Alcian blue, Picrosirius red, and Alizarin red stains of MSC microtissue constructs at day 2 and day 4 maturation levels seeded in the absence of the supporting hydrogel (μT only) and encapsulated in the OA (μT + Gel) after 6 weeks of culture. Note the positive calcium deposits in the microtissues constructs cultured in the absence of the supporting hydrogel (arrow). Collagen I, collagen II, and collagen X stains of MSC microtissues constructs at day 2 and day 4 maturation levels seeded in the absence of the supporting hydrogel (μT only) and encapsulated in the OA (μT + Gel) after 6 weeks of culture. (B) Total amount of DNA, sGAG/DNA, and collagen/DNA of MSC microtissue constructs at day 2 and day 4 maturation levels seeded in the absence of the supporting hydrogel (μT only) and encapsulated in the OA (μT + Gel) after 6 weeks of culture. Scale bar: 200 μm.

Next, the chondrogenic capacity of the microtissue-derived constructs was evaluated by histological and biochemical analyses after 6 weeks of culture (Figure 2). Constructs generated from day 2 and day 4 microtissues stained positive for sGAG in the presence and absence of the OA hydrogel, with more heterogeneous staining observed in the μT + Gel groups (Figure 2). The individual microtissues were more observable in the presence of the supporting gel, particularly in picrosirius stained sections, suggesting that the presence of OA may be delaying the fusion and/or the remodeling of the individual microtissues (Figure 2). Encapsulation in the OA hydrogel did not significantly affect overall levels of sGAG and collagen deposition. A trend toward higher GAG deposition was observed in constructs generated using day 2 microtissues compared to day 4 microtissues, with (*p* = 0.1398) and without (*p* = 0.4173) hydrogel encapsulation (Figure 2). No significant differences in DNA content were observed across the experimental groups. For comparison, DNA, GAG, and collagen values for native caprine articular cartilage are provided in Figure S-1.

All microtissue-derived constructs stained positive for type II collagen after 6 weeks of chondrogenic induction, although positive staining for type I collagen deposition was also observed (Figure 2). The day 4 μT only group also stained positive for type X collagen (Figure 2). Because of these findings, we decided to continue experimentation with day 2 microtissues.

### 3.2 Dynamic Culture Modulates Matrix Deposition by Microtissues Encapsulated within OA

**Figure 3:**
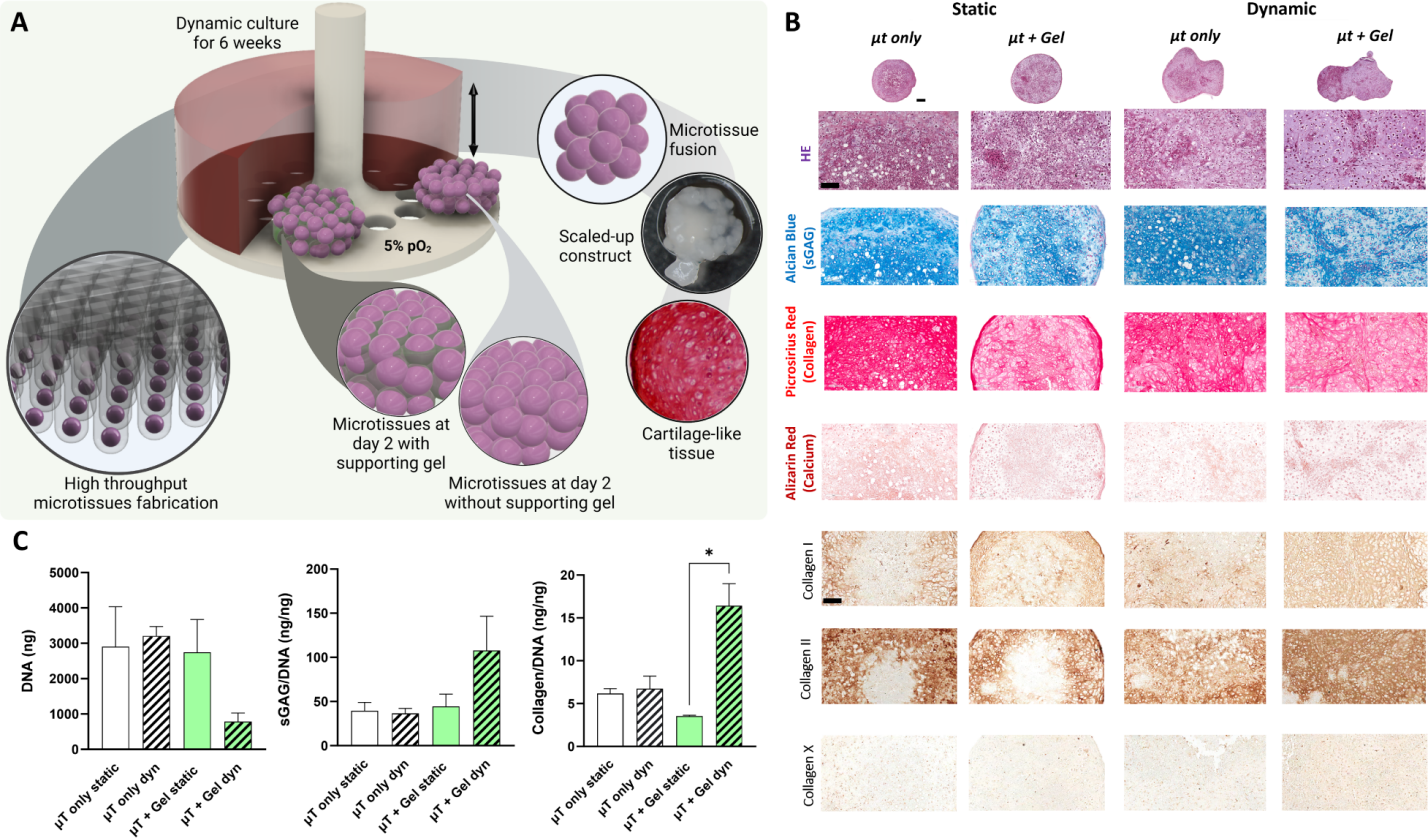
Chondrogenesis of MSC-derived microtissues in OA hydrogels in static and dynamic culture conditions. (A) All constructs were cultured in dynamic and static conditions and maintained in chondrogenic induction under physioxic conditions for up to 6 weeks. (B) Hematoxylin and eosin, Alcian Blue, picrosirius red, and alizarin red staining of the microtissue-derived constructs after 6 weeks of culture in static and dynamic conditions. (C) Quantification of DNA, sGAG/DNA, and collagen/DNA levels in static and dynamic conditions after 6 weeks of culture. The data are expressed as mean ± SD. The asterisks indicate *p*-values obtained by nonpaired *two-way* ANOVA followed by Tukey’s multiple comparisons post-test (*p < 0.05). Scale bars: top of (B)—400 μm; bottom of (B)—100 μm.

Generating large, homogeneous tissues using highly cellular microtissues is challenging due to their significant nutrient demands. This may partially explain, for example, the heterogeneous deposition of sGAGs observed in constructs generated in the presence of a temporary supporting hydrogel. In an attempt to address this concern, we next exposed the microtissue-derived constructs to dynamic culture conditions and compared outcomes to static culture conditions (Figure 3A). The histological analyses revealed positive staining for sGAG under both static and dynamic conditions in all groups (Figure 3B). Dynamic culture appeared to support the development of geometrically larger tissues with a minor increase in calcium deposition within the constructs (Figure 3B), although all groups stained weakly for type X collagen deposition. Dynamic culture did not appear to influence type I or type II collagen deposition. Following encapsulation in the OA hydrogel, dynamic culture appeared to reduce the total DNA content but increase collagen synthesis, as evidenced by a significant increase in collagen/DNA (*p* = 0.0150) (Figure 3C). No statistically significant changes in GAG accumulation were observed under dynamic culture conditions. Furthermore, dynamic culture was not found to alter DNA levels or collagen and GAG syntheses in the absence of the supporting OA hydrogel.

### 3.3 Microtissues Fuse and Generate a Cartilaginous Matrix following 3D Bioprinting in an OA Bioink

**Figure 4:**
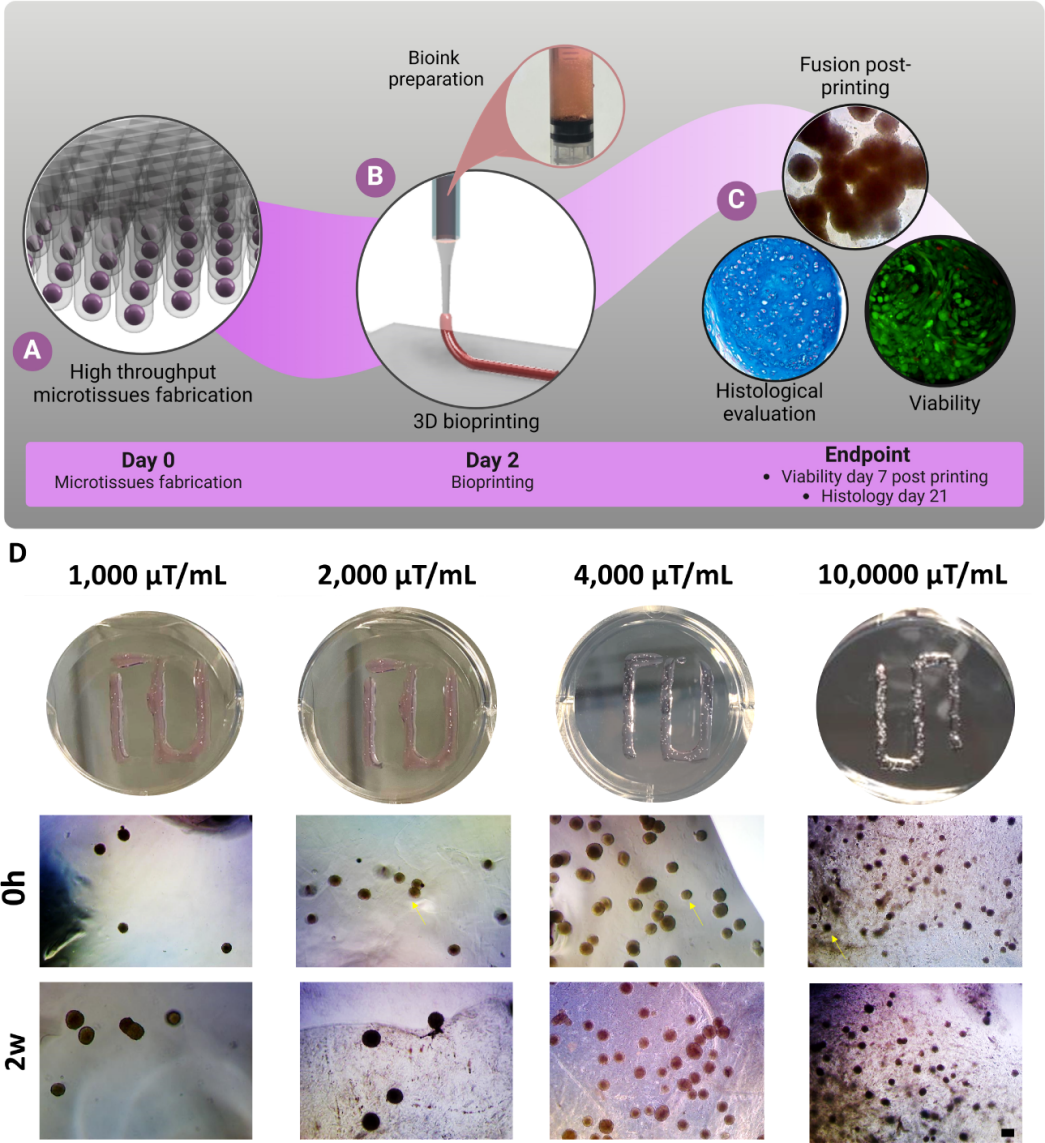
3D bioprinting of different densities of MSC-derived microtissues in an OA bioink. (A) Nonadherent agarose microwells were fabricated to produce up to 1,8,89 microtissues. (B) MSC microtissues were manually harvested from the nonadherent agarose microwells and encapsulated in the OA bioink in different densities for extrusion-based bioprinting. (C) The MSC microtissues were analyzed for fusion capacity, viability, and chondrogenic potential. (D) Macroscopic images of 1,000, 2,000, 4,000, and 10,000 microtissues/mL groups encapsulated in the OA bioink after the bioprinting process (arrows). Phase contrast images of 1,000, 2,000, 4,000, and 10,000 microtissues/mL groups at 0 h and 2 weeks after the bioprinting process. Note the different densities of microtissues within the OA bioinks after 2 weeks of culture (arrows). Scale bar: 200 μm.

We next explored the utility of OA as a bioink for supporting the 3D bioprinting of cartilage microtissues. To this end, OA was combined with 5% gelatin to generate a bioink with shear-thinning properties suitable for 3D (bio)printing (Figure S-3). In order to 3D bioprint the microtissues, we initially explored different densities of microtissues within the OA-based bioink and assessed the subsequent resolution of the extruded filaments (Figure 4). At a low density of 1,000 microtissues/mL of OA, the microtissues were easily extruded at a pressure of 35–40 kPa and a speed of 4 mm/s (Table 1). Macroscopically, it was possible to observe microtissues inside the OA filament after the bioprinting process (Figure 4D, arrow) and after 2 weeks by phase contrast (Figure 4D). At a density of 2,000 microtissues/mL, the microtissues were extruded using a pressure of 45–50 kPa and a speed of 4 mm/s (Table 1). The microtissues could again be visualized in the OA ink after the bioprinting process (Figure 4D, arrow) and after 2 weeks of culture (Figure 4D). At a density of 4,000 microtissues/mL of OA (Figure 4D), the microtissues were extruded at a pressure of 55–60 kPa and a speed of 4 mm/s (Table 1). The microtissues were still encapsulated in the OA filament after 2 weeks of culture, with some limited evidence of microtissue fusion (Figure 4D). At a density of 10,000 microtissues/mL of OA (Figure 4D), the microtissues were extruded at a pressure of 70 kPa and a speed of 5 mm/s (Table 1). The microtissues were still encapsulated in the OA filament after 2 weeks of culture (Figure 4D).

**Table 1:** Microtissue Density, Pressure, and Speed Parameters for Extrusion-Based Bioprinting

μT density (mL)	pressure (kPa)	speed (mm/s)
1,000	35–40	4
2,000	45–50	4
4,000	55–60	4
10,000	70	5

**Figure 5:**
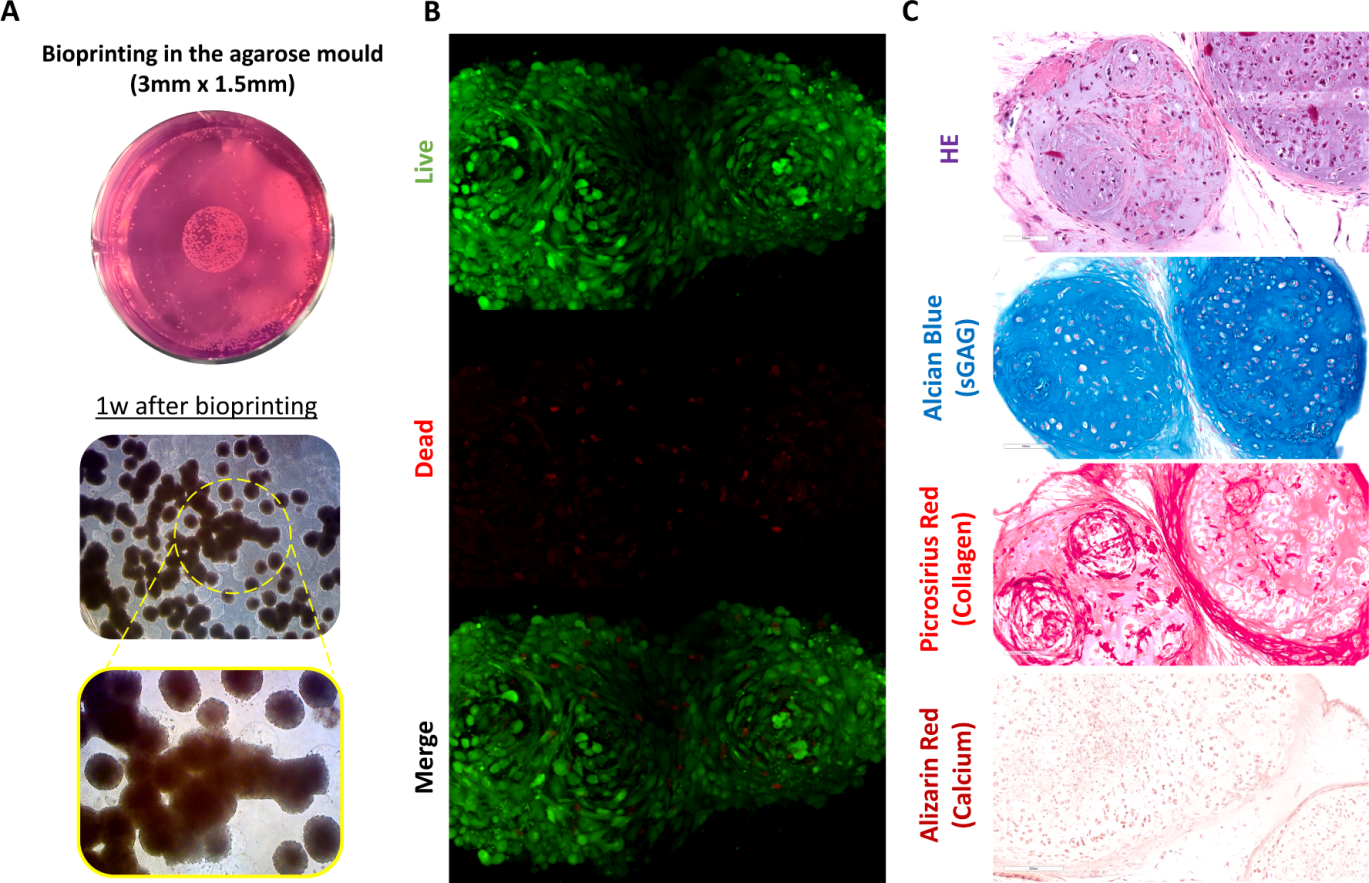
3D-bioprinted constructs show high viability and are positive for sGAG deposition after 6 weeks of chondrogenic induction. (A) Macroscopic view of the MSC microtissues directly after the bioprinting process and phase contrast of the constructs after 1 week of chondrogenic induction. Note the fusion of the microtissues in the bioink (yellow circle and magnification image in the yellow square). (B) Viability assessment of the constructs 10 days after the bioprinting process. Note that most of the construct was positive for live cells stained in green for Calcein-AM, with only low numbers of dead cells stained in red for EthD-1. (C) Hematoxylin and eosin, Alcian Blue, picrosirius red, and alizarin red stains of the bioprinted constructs after 6 weeks of chondrogenic induction. Scale bars: (A) 200 μm; (B) 100 μm; (C) 200 μm.

We next assessed chondrogenesis following the bioprinting of microtissues at the higher density of 10,000/mL of OA (Figure 5A). At 1-week postbioprinting, it was possible to observe multiple regions of microtissue fusion within the OA ink (Figure 5A, arrow). High levels of cell viability, with limited cell death, were observed (Figure 5B). After 6 weeks of chondrogenic induction, histological analyses revealed positive staining for sGAG and collagen (Figure 5C), with no calcium deposition (Figure 5C).

## 4 Discussion

In this work, we engineered microtissue-derived cartilage grafts using OA as a temporary supporting hydrogel or bioink. We observed that microtissue fusion is still possible following encapsulation into an OA hydrogel, albeit at levels that may be somewhat delayed, compared to those observed in the absence of this supporting hydrogel. Robust chondrogenesis was observed in the presence and absence of OA, with this supporting hydrogel enabling the engineering of cartilage grafts that undergo less contraction during culture. We then examined the suitability of OA as a supporting bioink for the 3D bioprinting of cartilaginous microtissues. Cells in the microtissues were viable after 3D bioprinting, and robust cartilage-specific ECM deposition was observed postprinting.

We first assessed the capacity of the microtissues at days 2 and 4 of “maturation” to fuse in the presence and absence of the supporting OA hydrogel. The fusion of microtissues is a biophysical process guided by cell-to-cell and cell-to-extracellular matrix interactions, allowing them to be used as biological building-blocks for tissue engineering applications.^33^ Previous studies have shown that early “maturation” levels lead to more homogeneous fusion of individual microtissues and to the formation of large and functional cartilage grafts.^11,34^ Here, we observed that microtissues encapsulated in the temporary OA hydrogel generated a hyaline-like cartilage graft over the 6-week culture period. While a number of previous studies have explored the fusion and chondrogenesis of microtissues encapsulated in more permanent hydrogels such as GelMA or chitosan,^12,35−38^ we believe the use of a rapidly degrading OA supports superior fusion of the encapsulated microtissues. Such OA hydrogels typically degrade over 2–3 weeks *in vitro*, enabling the engineering of *scaffold-free* cartilaginous tissues.^30,31^ It is perhaps unsurprising that even such a rapidly degrading hydrogel will impair fusion to some degree by acting as a physical barrier between individual microtissues. Clearly, a balance needs to be found between enabling fusion while simultaneously maintaining the desired shape of the overall construct.

Irrespective of their maturation levels (day 2 or 4) prior to encapsulation into OA, the microtissues were able to generate cartilage tissue rich in sGAG and type II collagen and were negative for calcium deposition. The sGAG content in such engineered tissues approached that measured in caprine articular cartilage, although collagen deposition was still lower than that found in the native tissue (Figure S-1). This agrees with previous studies that have demonstrated that microtissues are capable of generating sGAG-rich tissue following encapsulation into GelMA^36,37^ and other polymer-based hydrogels.^38,39^ In general, little evidence of hypertrophy was observed in the engineered tissues, with most constructs staining negative for collagen type X. There was some evidence of calcification of the tissues engineered in the absence of OA, although this was not consistently observed in all experimental replicates. TGF-β3 primed bone marrow-derived MSCs are known to progress to hypertrophy *in vitro*,^40−42^ so these findings are perhaps unsurprising.

It is well-known that oxygen levels together with nutrient availability within cell-rich constructs play a crucial role in regulating chondrogenesis of MSCs.^43,44^ Dynamic culture has been implemented in cartilage tissue engineering in an attempt to improve nutrient and oxygen transport within larger engineered constructs.^45−47^ Even relatively simple dynamic culture regimes, similar to that utilized here, have been shown to improve chondrogenesis of MSCs following hydrogel encapsulation.^20,48^ In addition, it has been shown that the mechanical microenvironment of MSCs in culture directly impacts extracellular matrix composition and the functionality of the resulting cartilage construct.^49^ We observed that microtissues encapsulated in OA and maintained in dynamic culture conditions appeared geometrically larger and secreted higher levels of collagen/DNA than those observed in static controls. Interestingly, dynamic culture did not alter matrix synthesis in the microtissue only group with no supporting OA hydrogel. This suggests that the OA might be supporting the retention of cell-secreted cartilage ECM components under dynamic culture conditions. It is also likely that a different mass transport and biophysical environment is generated in the OA group compared to the microtissue only group in the dynamic culture, which in turn could also benefit overall levels of chondrogenesis. Further studies are required to understand the mechanism (*e.g.*, matrix retention and/or altered synthesis) by which a supporting OA hydrogel might enhance matrix accumulation when engineering cartilaginous constructs using microtissues.

We also evaluated the potential of OA to be used as a bioink for extruding microtissues at different densities. The use of microtissues as building-blocks for 3D bioprinting applications is gaining increased traction in the field of biofabrication.^50^ In particular, the “*Kenzan*” method^51^ and the direct automatic deposition of microtissues in support hydrogels^52^ have successfully been used in the engineering of different tissue models. To the best of our knowledge, only a small number of studies have explored extrusion-based bioprinting of microtissues for musculoskeletal tissue engineering applications.^12,14,37^ Different challenges are associated with using microtissues in bioinks for extrusion-based bioprinting, including (1) the relatively large size and shape of microtissues, which increases the risk of needle clogging; (2) the risk of needle clogging increases when attempting to print a high density of microtissues; (3) ensuring a homogeneous distribution of microtissues inside the printed filament; and (4) ensuring fusion of the individual microtissues to form a cohesive tissue. Here, we observed that when the density of microtissues was increased, the printing parameters had to be changed to generate a consistent print. In particular, we found that the extrusion pressure had to be increased when printing higher microtissue densities to avoid clogging of the needle. Although we had to increase the extrusion pressure, the viability of the microtissues was not obviously impaired.

Microtissues were observed to fuse and undergo chondrogenesis when printed at sufficiently high densities. Moreover, we observed that when the microtissues were encapsulated in unmodified 3.5% (w/v) alginate (Figure S-2), the fusion was impaired, likely due to the persistence of the hydrogel between the microtissues. However, as the OA is a temporary support hydrogel, the fusion of microtissues was successful. De Moor and collaborators (2020)^12^ bioprinted MSC-derived microtissues using GelMA as a bioink for cartilage tissue formation. After the extrusion of the bioink, the authors observed that the microtissues were viable, were able to fuse, and stained positive for sGAG deposition. In accordance, we observed that the microtissues were viable 10 days after bioprinting, fused after 1 week, and deposited a tissue rich in sGAG and collagen over 6 weeks in chondrogenic culture conditions. Unlike other studies that only explored a relatively low density of microtissues during bioprinting,^14^ we also showed that it is possible to bioprint using different densities of microtissues in the OA bioink. This set of results suggests that OA-based bioinks support the bioprinting of microtissues and their capacity to undergo chondrogenesis.

## 5 Conclusion

In conclusion, we explored the potential of MSC-derived microtissues as biological building-blocks to biofabricate cartilage constructs. A key novelty of our approach is the use of a temporary supporting hydrogel (OA) to enable the engineering of a scaled-up graft using numerous cartilage microtissues. This hydrogel will rapidly degrade in culture,^30,31^ ideally leading to the development of a *scaffold-free* cartilage tissue that should be less likely to invoke a foreign-body response *in vivo*. We also demonstrated the utility of OA as a bioink to enable the extrusion-based bioprinting of microtissues at relatively high densities. The use of microtissues as biological units in 3D bioprinting may allow the biofabrication of more organized functional structures leading to better articular cartilage regeneration outcomes.

## Supporting Information Available

DNA, sGAG, and collagen of native goat articular cartilage (Figure S-1); microtissues at day 4 maturation level do not fuse in unmodified 3.5% alginate after 14 days of chondrogenic induction (Figure S-2); rheological characterization and 3D printing of 4% OA bioinks (Figure S-3) (PDF)
