# Supplementary material for: Rapidly Degrading Hydrogels to Support Biofabrication and 3D Bioprinting Using Cartilage Microtissues
Source: ACS Biomater Sci Eng. 2024 Sep 6;10(10):6441–50. doi: 10.1021/acsbiomaterials.4c00819 (PMC11480940; doi:10.1021/acsbiomaterials.4c00819)

Rapidly degrading hydrogels to support biofabrication and 3D bioprinting using cartilage microtissues

Gabriela S. Kronemberger[1,2,3], Francesca D. Spagnuolo[1,2,3], Aliaa S. Karam[1,2,3], Kaoutar Chattahy[1,2,3], Kyle J. Storey[1,2,3] and Daniel J. Kelly[1,2,3,4]

[1] Trinity Centre for Biomedical Engineering, Trinity Biomedical Sciences Institute, Trinity College Dublin, Dublin, Ireland.

[2] Department of Mechanical, Manufacturing and Biomedical Engineering, School of Engineering, Trinity College Dublin, Dublin, Ireland.

[3] Department of Anatomy and Regenerative Medicine, Royal College of Surgeons in Ireland, Dublin, Ireland.

[4] Advanced Materials and Bioengineering Research Centre (AMBER), Royal College of Surgeons in Ireland and Trinity College Dublin, Dublin, Ireland.

*Figure S-1: **DNA, sGAG and collagen of native goat articular cartilage.*** (A) DNA, (B) sGAG, (C) Collagen, (D) sGAG/DNA and (E) Collagen/DNA.

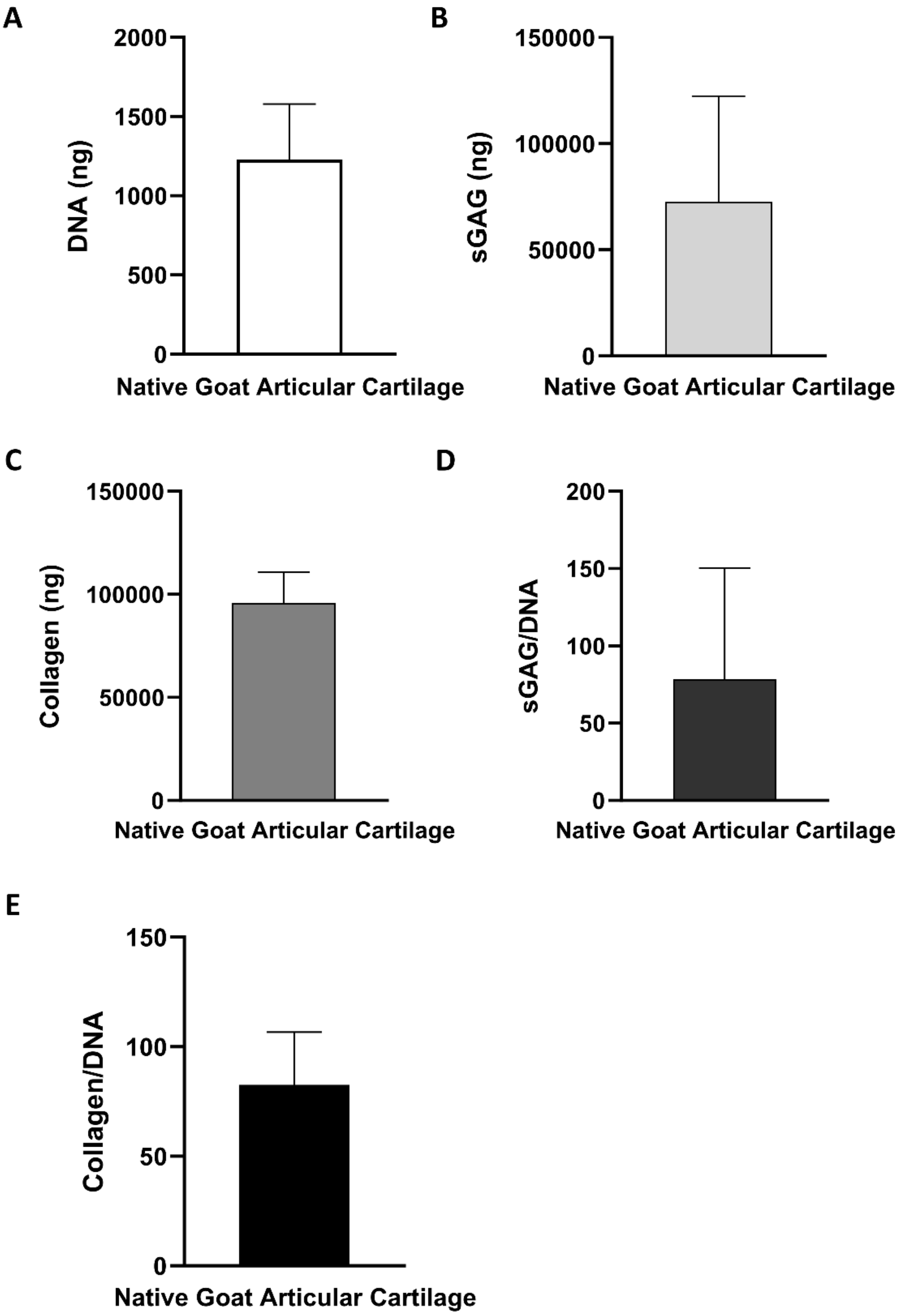

Figure S-2: Microtissues at day 4 maturation level do not fuse in unmodified 3.5% alginate after 14 days of chondrogenic induction. (A) Phase contrast images of MSC microtissues at day 4 maturation level at 0h, 24h, 72h and 7 days of culture in medium and high densities. (B) Hematoxylin and Eosin, Alcian Blue, Picrosirius Red and Alizarin red stains of the bioprinted constructs after 2 weeks of chondrogenic induction. Scale bars: (A) - 100 µm; (B) - 200 µm.

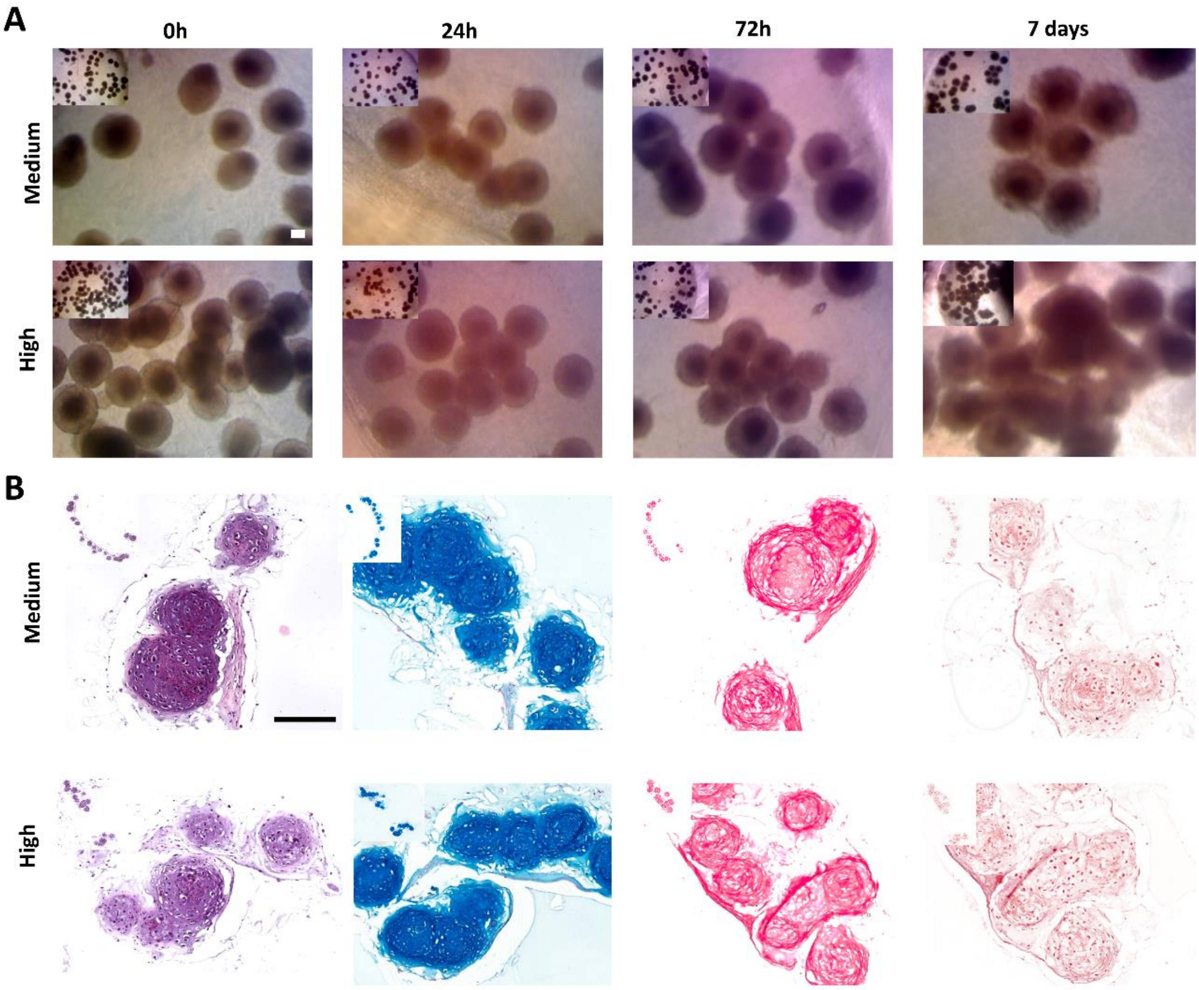

Figure S-3: Rheological characterization and 3D printing of 4% OA bioinks. (A) Viscosity of different bioinks and individual components investigated under a shear rate in the range of 0.1 to 1000 s-1. (B) Viscosity of different bioinks and individual components as a function of a temperature change from 4° C to 37° C. (C) Design and 3D printing of OA bioink prior and post crosslinking in 60 mM $CaCl_2$.

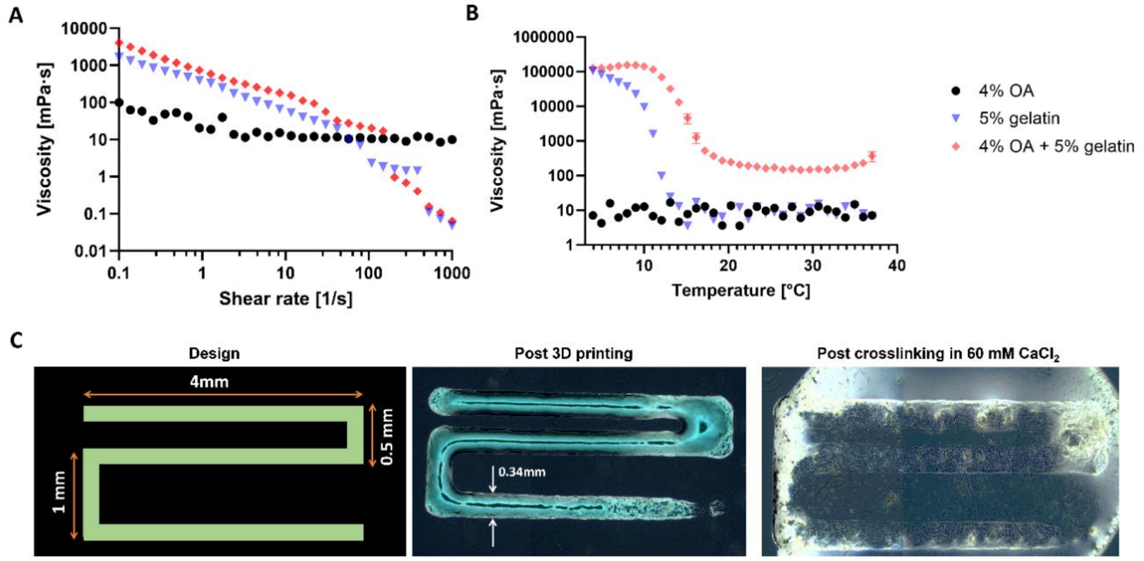

Supplement: Supplementary file 1 — ab4c00819_si_001.pdf [file ab4c00819_si_001.pdf]
